# Antioxidant Activity of Selected Stilbenoid Derivatives in a Cellular Model System

**DOI:** 10.3390/biom9090468

**Published:** 2019-09-09

**Authors:** Jakub Treml, Veronika Leláková, Karel Šmejkal, Tereza Paulíčková, Šimon Labuda, Sebastian Granica, Jaroslav Havlík, Dagmar Jankovská, Tereza Padrtová, Jan Hošek

**Affiliations:** 1 Department of Molecular Biology and Pharmaceutical Biotechnology, Faculty of Pharmacy, University of Veterinary and Pharmaceutical Sciences Brno, 61242 Brno, Czech Republic; 2 Department of Natural Drugs, Faculty of Pharmacy, University of Veterinary and Pharmaceutical Sciences Brno, 61242 Brno, Czech Republic; 3 Department of Pharmacognosy and Molecular Basis of Phytotherapy, Faculty of Pharmacy, Medical University of Warsaw, 02-097 Warsaw, Poland; 4 Department of Food Science, The Faculty of Agrobiology, Food and Natural Resources, The Czech University of Life Sciences Prague, 16500 Prague, Czech Republic; 5 Department of Chemical Drugs, Faculty of Pharmacy, University of Veterinary and Pharmaceutical Sciences Brno, 61242 Brno, Czech Republic

**Keywords:** stilbenoid, antioxidant, pro-oxidant, pyocyanin, Nrf2, macrophages

## Abstract

The stilbenoids, a group of naturally occurring phenolic compounds, are found in a variety of plants, including some berries that are used as food or for medicinal purposes. They are known to be beneficial for human health as anti-inflammatory, chemopreventive, and antioxidative agents. We have investigated a group of 19 stilbenoid substances in vitro using a cellular model of THP-1 macrophage-like cells and pyocyanin-induced oxidative stress to evaluate their antioxidant or pro-oxidant properties. Then we have determined any effects that they might have on the expression of the enzymes catalase, glutathione peroxidase, and heme oxygenase-1, and their effects on the activation of Nrf2. The experimental results showed that these stilbenoids could affect the formation of reactive oxygen species in a cellular model, producing either an antioxidative or pro-oxidative effect, depending on the structure pinostilbene (**2**) worked as a pro-oxidant and also decreased expression of catalase in the cell culture. Piceatannol (**4**) had shown reactive oxygen species (ROS) scavenging activity, whereas isorhapontigenin (**18**) had a mild direct antioxidant effect and activated Nrf2-antioxidant response element (ARE) system and elevated expression of Nrf2 and catalase. Their effects shown on cells in vitro warrant their further study in vivo.

## 1. Introduction

Stilbenoids are a group of secondary plant metabolites belonging to a wide family of plant phenolics. Although stilbenoids share a simple C6-C2-C6 unit, i.e., a 1,2-diphenylethylene structural unit-glycosylation, prenylation (including the formation of Diels-Alder adducts), and the ability to form benzofurans and oligomers make the group structurally large and diverse [1]. Historically, stilbenoids have been studied because of their prominent antibacterial, anticancer, and anti-inflammatory properties, and many studies have also described their antioxidant and chemopreventive properties. Among the stilbenoids chosen for these experiments (see Table 1) and also one of the best known is *trans*-resveratrol (**1**). This stilbenoid has been the focus of thousands of studies in recent years and has been shown to possess a promising potential for interactions with many cellular components. Its antiaging, anti-inflammatory, antioxidant, and immunomodulatory properties [2,3] have inspired clinical studies and *trans*-resveratrol (**1**) is currently described as a compound that is beneficial for the treatment of inflammatory diseases, metabolic syndrome, type 2 diabetes, and cardiovascular diseases. However, its relatively low bioavailability and rapid metabolism in vivo make the efficacy of this compound uncertain and reveal the need to investigate stilbenoid derivatives with greater activities [4].

Based on our previous results concerning anti-inflammatory activity (effect of lipopolysaccharide (LPS)-stimulated activation of nuclear factor κB/activator protein 1 (NF-κB/AP-1) and also subsequent signaling pathways) [3], we have tested 19 stilbenoid derivatives in the cell model THP-1-XBlue-CD14-MD2. We observed the production of reactive oxygen species (ROS) at the basal level and after stimulation with pyocyanin [5] after short- and long-termed exposure to determine any possible antioxidative and pro-oxidative effects, and compare its effects with quercetin [6]. These compounds that showed potential to interact with oxidative processes were further tested for their effects on the expression of enzymes involved in oxidative stress: catalase (CAT), glutathione peroxidase (GPx), heme oxygenase-1 (HO-1), superoxide dismutase 1 (SOD-1) and superoxide dismutase 2 (SOD-2). They were also tested for their effects on nuclear factor erythroid 2–related factor 2 (Nrf2), which controls the basal and induced expression of antioxidant response element–dependent genes to regulate cellular resistance to oxidative stress.

## 2. Materials and Methods

### 2.1. Test Compounds

The stilbenoid derivatives to be tested (**1–19**, Table 1) were isolated from natural sources or obtained commercially. The naturally occurring stilbenoids *trans*-resveratrol (**1**), pinostilbene (**2**), thunalbene (**3**), piceatannol (**4**), piceatannol-3’-*O*-β-glucopyranoside (**5**), batatasin III (**6**), pinostilbenoside (**7**), 1-(2,4-dihydroxyphenyl)-2-(4-hydroxyfenyl)-ethanone (**10**), 3,5-dimethoxystilbene (**11**), pterostilbene (**15**), pinosylvin monomethyl ether (**17**), and isorhapontigenin (**18**) were obtained from Sigma-Aldrich, synthetic compounds *trans*-stilbene (**12**), *cis*–stilbene (**13**), 4-stilbenecarboxylic acid (**14**), trans-α-methylstilbene (**16**), and 2,4,3´,5´-tetramethoxystilbene (**19**) were purchased from Sigma-Aldrich (Steinheim, Germany). The compounds 2-carboxyl-3-*O*-methyl-4´-β-d-glucopyranosyl-dihydroresveratrol (**8**) and 3-*O*-caffeoyl-(9→5)-β-apiosyl-(1→6)-β-glucopyranosyl-5,3´-*O*-dimethyldihydropiceatannol (**9**) were kindly provided by Dr. Sebastian Granica (Medical University of Warsaw, Warsaw, Poland) who had isolated them from *Tragopogon tommasinii* Sch.Bip. (Asteraceae, Cichorieae) [7].

### 2.2. Induction of Lipid Peroxidation

Lipid peroxidation was measured using linoleic acid and 2,2´-azobis(2-amidinopropane)dihydrochloride (AAPH) as has been previously described [8], with some slight modifications. The reaction mixture and also the positive control consisted of 8 mM linoleic acid (Sigma Aldrich, Saint Louis, MO, USA) and 20 mM of AAPH (Sigma Aldrich). Each test compound was added to an experimental tube at a concentration of 15 µM. The negative control contained only the vehicle (DMSO). The reaction mixtures were then incubated for 24 h at 37 °C. Following incubation, the content of malondialdehyde (MDA) formed by lipid peroxidation was measured as described in Section 2.2.1. All of the experiments were performed in triplicate.

#### 2.2.1. Thiobarbituric Acid Reactive Substances Assay

The content of MDA in each reaction mixture was quantified as described by Vasantha Rupasinghe and Yasmin [9], with some modifications. The thiobarbituric acid (TBA) reagent (20% (*w*/*v*) trichloracetic acid (Sigma Aldrich) along with 0.375% (*w*/*v*) TBA in 0.25M HCl (Sigma Aldrich)) was added to each reaction mixture. The mixtures were incubated for 30 min at 95 °C and then cooled to room temperature. The MDA-TBA adduct which formed was extracted from the mixture with an equal volume of butanol (Sigma Aldrich). The extraction was done in two steps: The mixtures were vigorously vortexed for 15 min, and then centrifuged for 5 min at 16,000× *g*. Each butanol fraction was transferred to a 96-well plate, and the absorbance was measured at 532 nm using a FluoStar Omega spectrophotometer (BMG Labtech, Ortenberg, Germany).

### 2.3. Cell Culturing

THP-1-XBlue-MD2-CD14 cells were supplied by Invivogen (San Diego, CA, USA) and cultured in RPMI 1640 medium containing stabilized 2 mM l-glutamine (Biosera, Nuaille, France), supplemented with antibiotics (100 U/mL penicillin and 100 mg/mL streptomycin (Biosera), and 10% fetal bovine serum (FBS) (HyClone, Logan, UT, USA). The cells were kept in an incubator at 37 °C in a water-saturated atmosphere of air containing 5% CO_2_. The suspensions of THP-1-XBlue-MD2-CD14 cells were passaged approximately twice a week.

The HepG2 human hepatoma cell line was purchased from the European Collection of Cell Cultures (Salisbury, UK). Cells were grown in DMEM low glucose medium (Biosera) supplemented with antibiotics (100 U/mL penicillin, 100 mg/mL streptomycin), 10% FBS, and 2 mM l-glutamine. Cultures were kept in an incubator at 37 °C in a water-saturated atmosphere of air containing 5% CO_2_. Stabilized cells (12−35th passage) were split into microtitration plates and used for further experiments.

All procedures, such as viability control (each time only cells with viability greater than 95% were used), erythrosine B staining, and light microscopy, were done in standard aseptic conditions. Each experiment for each compound was done three times in an independent triplicate.

### 2.4. Antioxidant Activity Testing

Antioxidant activity was initiated by triggering the formation of ROS by applying pyocyanin to the cultured cell. In order to measure the fluorescent probe 2´,7´-dichlorodihydrofluorescein diacetate (DCFH-DA) was introduced to the cell culture, where it was immediately deesterified to form 2´,7´-dichlorodihydrofluorescein, which then causes fluorescence when it was oxidized by ROS [10]. The compounds and standard of quercetin [6] (Koch Light Laboratories, Haverhill, UK) were tested at a final concentration of 2 μM, which previous cytotoxicity tests revealed as non-toxic for all of these compounds [3]. Stock solutions (20 mM) of the test compounds were prepared by dilution with DMSO, and stored frozen at −80 °C. Before each experiment, they were thawed and diluted to achieve the desired final concentration of 2 μM. Pyocyanin was used to trigger oxidative stress in the cells [5]. The stock solution of it at a concentration of 100 mM in DMSO was stored in a freezer. Before each experiment, the stock solution was thawed and diluted to achieve a final concentration of 100 μM per well. The maximum amount of DMSO in solution with cell culture was 0.1% (*v*/*v*).

#### 2.4.1. Determination of Antioxidant Activity

A suspension of cells in RPMI 1640 serum-free medium at a concentration of 500,000 cells/mL was aliquoted into a 96-well microtiter plate, 100 μL per well. The cells were incubated for 2 h at 37 °C in 5% CO_2_ atmosphere to acclimatize, and the test compound of final concentration 2 μM was added. Quercetin was used as the reference compound, along with a negative control—DMSO alone. This procedure was followed by 1 h of incubation (37 °C, an atmosphere of 5% CO_2_). Pyocyanin solution (as the positive control) was then added to all wells after that period, and the incubation followed next 30 min. After that DCFH-DA solution was added and after 30 min for the short term or 24 h for the long-term exposure, the fluorescence (excitation at 485 nm; emission at 538 nm) was measured using a FluoStar Omega spectrophotometer.

#### 2.4.2. Determination of Antioxidant Activity—Pyocyanin Free Model

The testing was carried out under the same conditions as were used to determine the antioxidant activity of the test compounds alone, without pyocyanin added to stimulate the formation of ROS. Pyocyanin served as a positive control for comparison. Both 2 h and 24 h exposures of cells to tested compounds were used.

### 2.5. Protein Expression of Antioxidant Enzymes

The effect of test compounds on protein expression of antioxidant enzymes was observed in THP-1-XBlue-MD2-CD14 cells. The cells were incubated in the form of floating monocytes (1,000,000 cells/mL) in 3 mL of a serum-free RPMI 1640 medium and seeded into 6-well plates in triplicate at 37 °C. After a 6 h treatment with one of the test compounds at a concentration of 2 μM dissolved in DMSO, the cells were collected using lysis buffer (50 mM Tris-HCl (pH 7.5), 1 mM EGTA, 1 mM EDTA, 1 mM sodium orthovanadate, 50 mM sodium fluoride, 5 mM sodium pyrophosphate, and 0.27 M sucrose) with protease inhibitors (Roche, Mannheim, Germany). The protein concentration was measured using a Bradford method protein assay kit (Sigma Aldrich) according to the manufacturer’s instructions.

To separate the proteins, 20 μg of the proteins from the cell lysates were loaded onto a 12% SDS-polyacrylamide gel. They were then transferred electrophoretically to polyvinylidene fluoride (PVDF) membranes with 0.2 µm pores (Bio-Rad, Hercules, USA) that were subsequently blocked using 5% bovine serum albumin (BSA) (SERVA, Heidelberg, Germany) dissolved in TBST buffer (10 mM Tris-HCl (pH 7.5), 150 mM NaCl, and 0.1% (*v*/*v*) Tween-20) for 1 h.

The membranes were incubated with the primary antibody (mouse anti-CAT 1:1000 (Sigma-Aldrich; product No. C0979), rabbit anti-SOD1 1:1000 (Sigma-Aldrich; product No. HPA001401), rabbit anti-SOD2 1:1000 (Abcam, Cambridge, UK; product No. ab16956), rabbit anti-NRF2 1:1000 (Abcam; product No. ab137550), rabbit anti-GPx1 (Abcam; product No. ab22604), mouse anti-HO-1 (Abcam; product No. ab13248), or mouse anti-β-actin 1:5000 (Abcam; product No. ab8226)) at 4 °C overnight. After washing, the secondary antibody (anti-mouse IgG (Sigma-Aldrich; product No. A0168) or anti-rabbit IgG (Sigma-Aldrich; product No. A0545) at a dilution of 1:2000), was applied to the membranes, and they were incubated for 1 h at room temperature. Bands were visualized using a chemiluminescent kit (Bio-Rad) and a PXi Syngene Chemiluminescent Imaging System (Syngene, Cambridge, UK) and quantified by optical densitometry (AlphaEaseFC 4.0.0 software, Alpha Innotech, San Leandro, CA, USA).

### 2.6. Activation of Nrf2-Antioxidant Response Element System

The influence of the test compounds on the activity of Nrf2 was estimated using an antioxidant Response Element (ARE) reporter kit (BPS Bioscience, San Diego, CA, USA). HepG2 cells were transiently transfected for 24 h (35,000 cell/well in 96-well plates) with the ARE luciferase reporter vector (firefly luminescence) plus a constitutively expressing Renilla vector using the TransFast Transfection reagent (Promega, Madison, WI, USA). After serum recovery, the cells were treated for 24 h with each of the test compounds at a concentration of 2 μM dissolved in DMSO (a non-toxic concentration). As a positive control for this experiment, we have used DL-sulforaphane (Sigma Aldrich) at a concentration of 10 µM solved in DMSO, as recommended by ARE reporter kit. The luciferase activity from the cell lysates was detected using a Dual luciferase reporter assay system (Promega) and a FluoStar Omega spectrophotometer. Data were normalized to Renilla luminescence.

### 2.7. Statistical Evaluation

The experimental data were processed in Excel (Microsoft). The results of the blank experiment were subtracted, and the experimental results were normalized to the positive control. Outliers were removed using the ROUT statistical method (Q = 5%) in GraphPad Prism 6.01 (San Diego, CA, USA). Groups were compared with the help of the one-way ANOVA test followed by Fisher’s LSD multiple comparison test. The value *p* < 0.05 was assigned as statistically significant.

## 3. Results

The antioxidant or pro-oxidant activity of 19 natural and synthetic stilbenoids was determined using various in vitro methods. First of all, the influence of test compounds on lipid peroxidation in a cell-free assay was measured. Lipid peroxidation was studied because lipids are the main components of cellular membranes and often the targets of oxidative stress. The products of this oxidation are lipid peroxides, which can have toxic effects on other cellular components, such as DNA or proteins [11].

**Figure 1:**
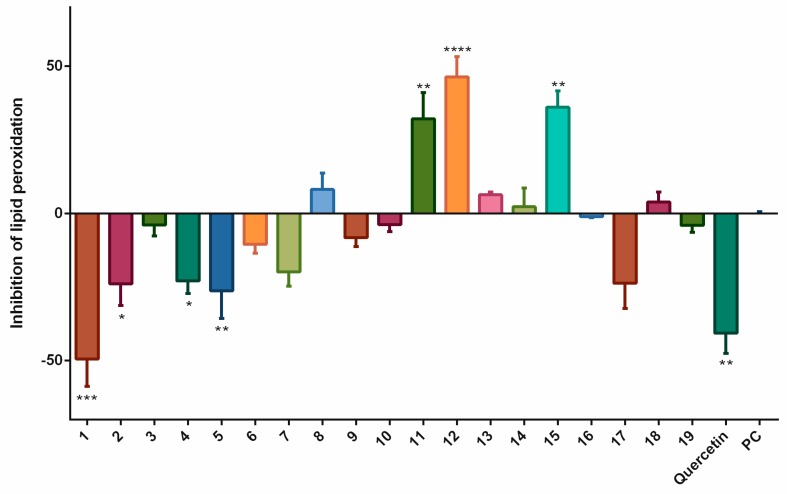
The effects of stilbenoids **1–19** (at a concentration of 15 μM) on the lipid peroxidation of linoleic acid caused by 2,2´-azobis(2-amidinopropane)dihydrochloride (AAPH), and measured as the production of malondialdehyde (MDA) using the thiobarbituric acid reactive substances (TBARS) assay. Quercetin was used as a standard (15 μM) and AAPH alone served as the positive control (PC). The negative control (NC) contained linoleic acid alone, and thus, no lipid peroxidation occurred. The effects of the vehicle were subtracted from that of each stilbenoid. * = *p* < 0.05; ** = *p* < 0.01; *** = *p* < 0.001; and **** = *p* < 0.0001.

Thus, we evaluated the effects of stilbenoids in the peroxidation of lipids in linoleic acid by AAPH. The results are displayed in Figure 1. *Trans*-stilbene (**12**) with 46.4% inhibition of lipid peroxidation, followed by pterostilbene (**15**) and 3,5-dimethoxystilbene (**11**), most effectively diminished lipid peroxidation. On the other hand, quercetin did not inhibit lipid peroxidation. This was rather surprising because quercetin had been chosen as the standard precisely because we did not expect it to show pro-oxidant activity at a concentration of 15 µM [12]. Resveratrol (**1**) with −49.4% was the most effective pro-oxidant stilbenoid. Pinostilbene (**2**), piceatannol (**4**), and piceatannol-3´-*O*-β-glucopyranoside (**5**) also showed some statistically significant pro-oxidant effects.

**Figure 2:**
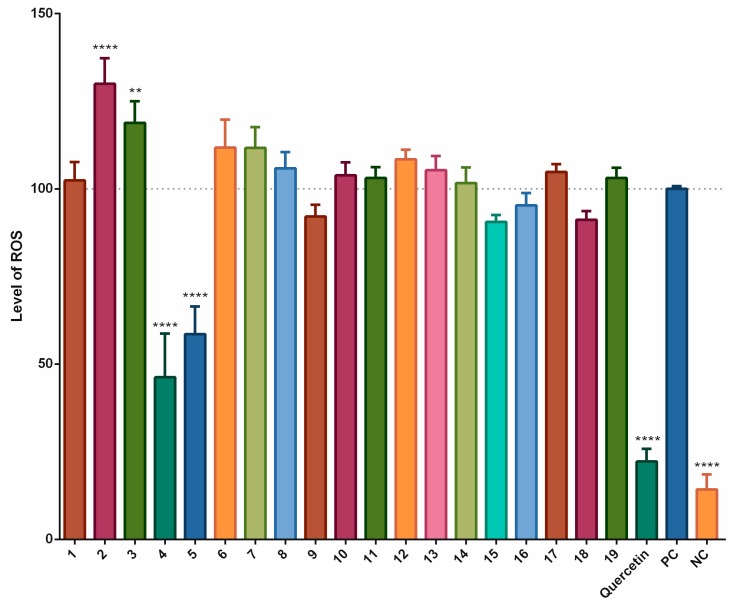
Antioxidant and pro-oxidant effects of stilbenoids **1–19** (at a concentration of 2 μM) on the formation of ROS after 1 h of incubation. In the THP-1-XBlue-CD14-MD2 cell model, the formation of ROS was triggered by adding 100 μM pyocyanin; quercetin was used as the standard (2 μM), pyocyanin alone served as the positive control (PC; 100 μM) and the vehicle alone was the negative control (NC). ** = *p* < 0.01; **** = *p* < 0.0001.

To evaluate these results, we have employed an antioxidant method using the THP-1-XBlue-CD14-MD2 cell model. Some stilbenoids showed an effect on the pyocyanin-stimulated formation ROS after 1 h of incubation. A statistically significant decrease in the levels of ROS was observed for piceatannol (**4**) (53.8%), and piceatannol-3´-*O*-β-glucopyranoside (**5**) (41.4%), but neither compound was as active as the quercetin standard (77.8%). Several other compounds showed some increase in the formation of ROS. For pinostilbene (**2**) and thunalbene (**3**), this effect was statistically significant, showing them to act as pro-oxidants in this short-term incubation model (Figure 2).

From the results, we can observe that the stilbenoids which acted as antioxidants in lipid peroxidation assay (e.g., *trans*-stilbene (**12**)) did not prove to be antioxidants in a cell-based assay. The advantage of assays using cell cultures is that apart from observing the activity, we can also have a clue whether the compound is able to cross the cell membrane or not.

**Figure 3:**
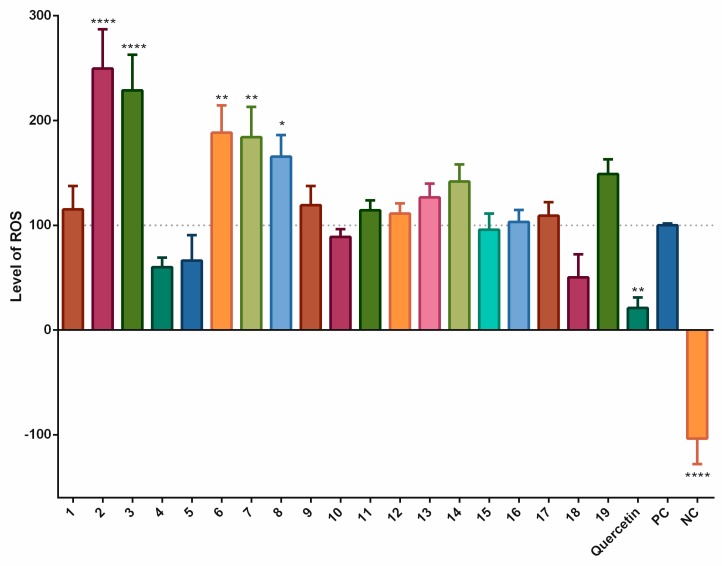
Antioxidant or pro-oxidant effects of stilbenoids **1–19** (at a concentration of 2 μM) on the formation of ROS after 24 h of incubation. In the THP-1-XBlue-CD14-MD2 cell model, the formation of ROS was triggered by adding 100 μM pyocyanin; quercetin was used as the standard (2 μM), pyocyanin alone served as the positive control (PC; 100 μM) and the vehicle alone was the negative control (NC). * = *p* < 0.05; ** = *p* < 0.01; and **** = *p* < 0.0001.

The effects of long-termed incubation of stilbenoids **1–19** on the formation of ROS following simulation with pyocyanin were evaluated using a 24 h period of incubation. Figure 3 shows that, as in the short-termed system, piceatannol (**4**), and piceatannol-3´-*O*-β-glucopyranoside (**5**) as did isorhapontigenin (**18**) decreased the formation of ROS, but a statistically significant effect was observed only for quercetin, the positive control. In contrast, pinostilbene (**2**), thunalbene (**3**), batatasin III (**6**), pinostilbenoside (**7**), and 2-carboxyl-3-*O*-methyl-4´-*β*-D-glucopyranosyl-dihydroresveratrol (**8**) increased the formation of ROS to a statistically significant degree.

**Figure 4:**
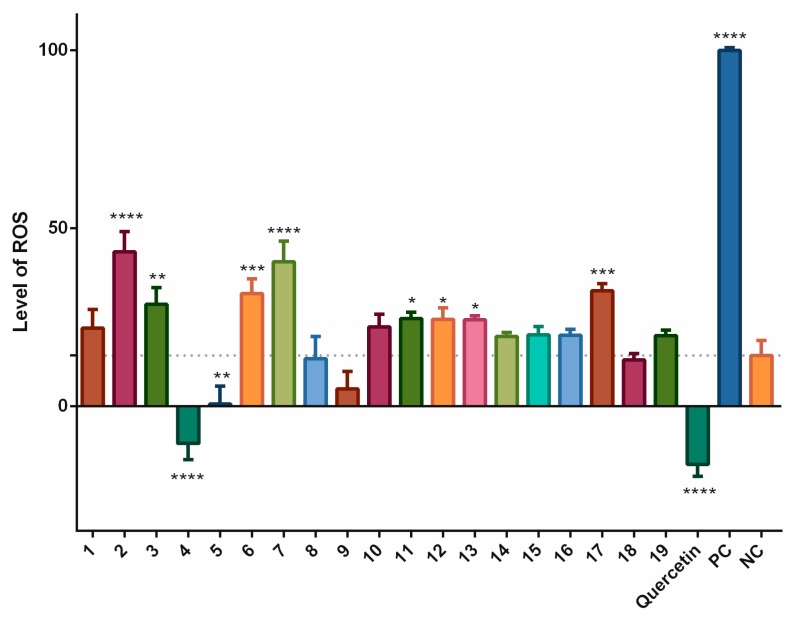
Antioxidant and pro-oxidant effects of stilbenoids **1–19** alone (at a concentration of 2 μM) on the formation of ROS after 2 h of incubation. In the THP-1-XBlue-CD14-MD2 cell model, the formation of ROS was triggered by stilbenoids alone; quercetin was used as the standard (2 μM), pyocyanin alone served as the positive control (PC; 100 μM), and the vehicle alone was the negative control (NC). * = *p* < 0.05; ** = *p* < 0.01; *** = *p* < 0.001; and **** = *p* < 0.0001.

We also evaluated the effects of the stilbenoids alone in the cell model, without artificially stimulating the production of ROS. Incubation times of 2 h and 24 h were chosen. The results of the 2-h incubation are shown in Figure 4. The pattern was similar to that for the data shown in Figure 1. Statistically significant increases in the levels of ROS were observed for pinostilbene (**2**) (29.1%), batatasin III (**6**) (17.4%), pinostilbenoside (**7**) (26.3%), and pinosylvin monomethyl ether (**17**) (18.2%) compared to the negative control. Thunalbene (**3**), 3,5-dimethoxystilbene (**11**), *trans*-stilbene (**12**), and *cis*-stilbene (**13**) were also pro-oxidant, but with less significant increases in production of ROS. On the other hand, piceatannol (**4**), piceatannol-3´-*O*-β-glucopyranoside (**5**), and quercetin standard significantly reduced the levels of ROS.

**Figure 5:**
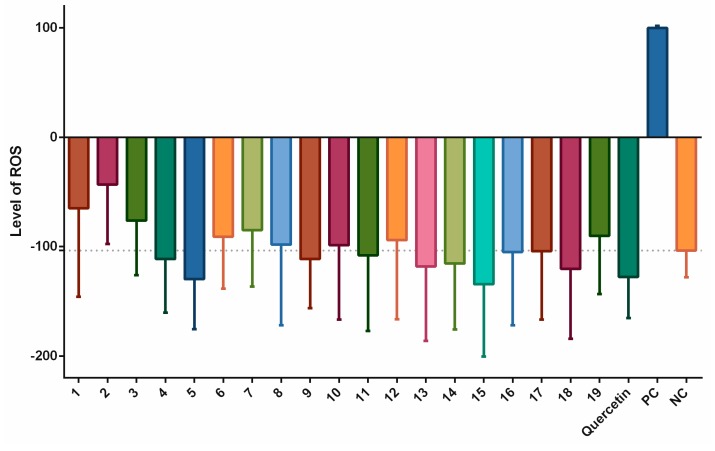
Antioxidant and pro-oxidant effects of stilbenoids **1–19** alone (at a concentration of 2 μM) on the formation of ROS after 24 h of incubation. In the THP-1-XBlue-CD14-MD2 cell model, the formation of ROS was triggered by stilbenoids alone; quercetin was used as the standard (2 μM), pyocyanin alone served as the positive control (PC; 100 μM), and the vehicle alone was the negative control (NC).

Results of the 24 h incubation are shown in Figure 5. We detected only slightly decreased levels of ROS after incubation with some of the stilbenoids alone, but, a pronounced increase was observed for resveratrol (**1**) (36.9%) and pinostilbene (**2**) (60.4%) compared to the negative control.

**Figure 6:**
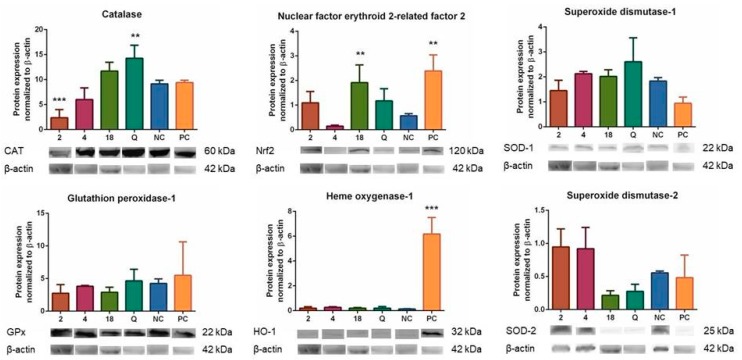
Effects of compounds **2**, **4**, and **18** (at a concentration of 2 µM) on the levels of selected antioxidant enzymes CAT, GPx, HO-1, and SOD-1 and -2, and on the expression of Nrf2 after 6 h of incubation. The THP-1-XBlue-CD14-MD2 cell model was used with quercetin as the standard (2 μM), pyocyanin alone (100 µM) as the positive control (PC), and the vehicle alone as the negative control (NC). ** = *p* < 0.01; *** = *p* < 0.001; and **** = *p* < 0.0001.

Based on the of the results described above, we analyzed the effects of compounds **2**, **4**, and **18** on the expression of enzymes associated with oxidative stress, because the observed activities might also have been caused by effects of these compounds on endogenous enzymatic antioxidant systems. We analyzed the effects of compounds **2**, **4**, and **18** at a concentration of 2 µM after 6 h of incubation with THP-1-XBlue-CD14-MD2 cells on the levels of CAT, GPx, HO-1, SOD-1, and SOD-2. As shown in Figure 6, the test compounds did affect the expression of these enzymes. A 6 h of incubation with compound **2** had decreased the expression of CAT in a statistically significant manner (*p* < 0.001). However, the expression of the same enzyme (CAT) was significantly increased by quercetin (*p* < 0.01). Also, the expression of CAT enzyme was increased by compound **18**, but not in a significant manner. Compound **2** had proven to be pro-oxidant in the previous experiments, so it was not surprising that it downregulated the levels of antioxidant enzymes. On the other hand, quercetin and compound **18** had proven to be antioxidant and upregulated the expression of CAT.

For another protein expression, the results were significant when cells were incubated with compound **18** and positive control (*p* < 0.01) for Nrf2 protein. Also, the positive control pyocyanin significantly elevated the expression of HO-1 protein (*p* < 0.001). The changes in the results for other compounds and enzymes were not statistically significant, although expression of SOD-2 was elevated after incubation with compounds **2** and **4**.

**Figure 7:**
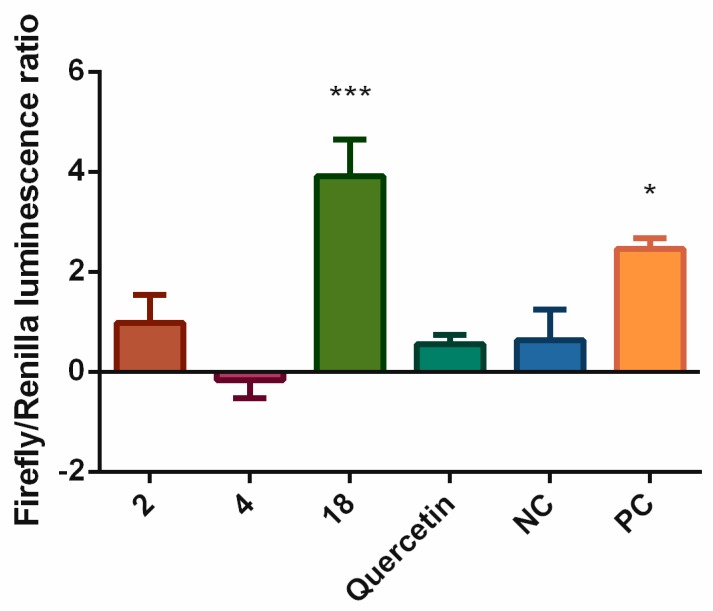
The effects of selected stilbenoids **2**, **4**, and **18** (at a concentration of 2 μM) on the activation of Nrf2-ARE system. The HepG2 cell model was transiently transfected with the ARE luciferase reporter vector firefly luminescence and a constitutively expressing Renilla vector. The results are expressed as the ratio of firefly to Renilla luminescence. Quercetin was used as the standard (2 μM), DL-sulforaphane was used as a positive control at a concentration of 10µM (PC), and the vehicle alone served as the negative control (NC). * = *p* < 0.05; *** = *p* < 0.001.

The obvious effects of compounds **2**, **4**, **18** and quercetin on the expression of several enzymes associated with antioxidant defense encouraged us to analyze their effects on the upstream regulator Nrf2. To verify the mechanism causing the effects of test compounds, we evaluated their direct effects on the activation of Nrf2-ARE. Figure 7 shows that compound **18**, as well as DL-sulforaphane, the positive control, triggered the activation of the Nrf2-ARE system in a statistically significant manner (*p* < 0.001 and *p* < 0.05, respectively). This might explain the mechanism behind the increased expression of Nrf2 and CAT induced by compound **18**. Surprisingly, quercetin did not activate the Nrf2-ARE pathway, although it was able to increase the expression of CAT.

## 4. Discussion

The antioxidant and pro-oxidant activities of natural and synthetic compounds can be tested in different ways. Cell-based methods have an advantage over biochemical screening in that they are more complex, and their results offer a more thorough interpretation of the conditions within the organism. We, therefore, used first the lipoperoxidation cell-free measurement of TBARS and then for verification the THP-1-XBlue-CD14-MD2 cell model and the formation of ROS triggered using pyocyanin (a blue-green phenazine pigment with oxidoreductive properties). It stimulates oxidative metabolism, increasing the formation of ROS via the oxidation of NADPH [5]. The flavonoid quercetin was used for reference because it is a well-known antioxidant, and at greater concentrations shows pro-oxidant properties [6]. Like the flavonoids represented by quercetin, the stilbenoids are phenolics found at high concentrations in medicinal plants, vegetables, walnuts, and edible fruits, such as grapes or berries [1]. The most well-known stilbenoid is resveratrol (**1**). In combination with anthocyanins, this compound is believed to be responsible for the beneficial phenomenon called the French paradox. In addition to its anti-inflammatory and antioxidant activities, it is antiviral, neuroprotective, cardioprotective, and chemoprotective [13].

Stilbenoids affect organisms in different ways, depending on their structural characteristics. Therefore, we studied a large group of synthetic and natural stilbenoids (**1–19**), to find out, if and how their structures affect their antioxidant or pro-oxidant effects in cellular systems. In the cell-free lipoperoxidation inhibition assay, the most active compound was *trans*-stilbene (**12**), followed by pterostilbene (**15**) and 3,5-dimethoxystilbene (**11**). And unexpectedly, quercetin and resveratrol (**1**) acted as pro-oxidants.

The cell-based antioxidant assay showed rather different outcomes. Piceatannol (**4**) and piceatannol-3´-*O*-β-glucopyranoside (**5**) showed significant antioxidant activities after a short-term incubation with cells stimulated by pyocyanin. 3-*O*-caffeoyl-(9→5)-β-apiosyl-(1→6)-β-glucopyranosyl-5,3´-*O*-dimethyldihydropiceatannol (**9**), pterostilbene (**15**), trans-α-methylstilbene (**16**) and isorhapontigenin (**18**) decreased the formation of ROS, but not significantly. The other compounds did not decrease the formation of ROS as much as quercetin, the positive control. On the contrary, pinostilbenoside (**7**) and pinostilbene (**2**) showed significant pro-oxidant activities. Clearly, the glycosylation of **4** reduced the antioxidant activity, however not significantly.

Long-term exposure of cells to compounds **1**–**19** with pyocyanin showed no statistically significant antioxidant effects, but isorhapontigenin (**18**) diminished the formation of ROS by 49.7%, followed by piceatannol (**4**) and piceatannol-3´-O-β-glucopyranoside (**5**). Mild activity (10%), greater than in the short-term exposure, was also observed for 1-(2,4-dihydroxyphenyl)-2-(4-hydroxyphenyl)-ethanone (**10**). Other compounds had little effects on the levels of ROS, or were pro-oxidative, as seen for pinostilbene (**2**), thunalbene (**3**), batatasin III (**6**), pinostilbenoside (**7**), and 2-carboxyl-3-*O*-methyl-4´-β-D-glucopyranosyl-dihydroresveratrol (**8**).

Twelve of the 19 test compounds are hydroxystilbenoids (with a free hydroxyl). These can be divided into two groups according to their structure: Those which can form quinoid systems after two-electron oxidation, and those which cannot [14]. Hydroxystilbenes that contain a catechol or pyrogallol moiety belong to the first group, whereas the ones which possess only phenol or resorcinol arrangements cannot be so oxidized to form quinoids [15]. Compounds in the first group generally display much greater scavenging effects and can increase their overall antioxidant activity, for example, by chelating metal ions [16]. Piceatannol (**4**) was the only compound with a free catechol moiety. The structurally similar piceatannol-3´-*O*-β-glucopyranoside (**5**) has an *ortho* hydroxy group, but the critical is occupied by a glycosidic bond. Compound **4** had a greater antioxidant activity, which confirms previous studies showing the antioxidant potential of highly hydroxylated stilbenoids [14,15]. Piceatannol (**4**) affects the same molecular targets involved in antioxidant defense (AP-1, Nrf2, HO-1, COX, iNOS, NF-κB, and IKK) as resveratrol (**1**), but it showed a much greater direct scavenging effect and also greater inhibition COX-2, NF-κB and the formation of the pro-inflammatory cytokines TNF-α and IL-1β [17,18]. Because the antioxidant activity is greater for the short-term exposure of cells than for the long, the direct scavenging effect will likely predominate over stimulation of the antioxidant defense, but possible improvement in the overall antioxidant status cannot be excluded [19,20]. The blocked hydroxy groups of stilbenoids may be responsible for reducing the antioxidant effect not only directly, but also indirectly, by reducing the ability to inhibit enzymes responsible for the formation of ROS, such as xanthinoxidase, monoaminoxidase or, - in case of nitrogen-containing reactive species - iNOS [21,22].

Another compound with antioxidant activity was isorhapontigenin (**18**), which showed only a small effect after one hour of incubation (8.8%), but much more (49.7%) after 24 h. This result can be attributed to modulation of the antioxidant defense by antioxidant or pro-oxidant enzymes. Studying the same compound, Abbas et al. have found increased levels of glutathione and the antioxidant enzymes SOD and CAT [23], and another study has shown the increased expression and activity of SOD-2 and GPx1 [24], which we also found.

Several test compounds were inactive, showing neither antioxidant nor pro-oxidant effects. Although other studies have reported antioxidant activities for these compounds, possible because greater concentration (5–50 µM) were tested [1,14,16], we feel concentrations for greater than those likely to be found are not realistic for the in vivo evaluation of biological effects.

Several studies have depicted stilbenoids as pro-oxidative compounds [14,16]. Resveratrol (**1**) and its hydroxylated derivatives can be oxidized either enzymatically or non-enzymatically to form phenoxy radical, which can, escaping further cellular antioxidant processing, increase levels of ROS. Pro-oxidative effects of hydroxylated stilbenoids have usually been described for much greater concentrations than were used in our study, but we observed pro-oxidant effects for several compounds at a concentration of 2 μM only for both short-term and long-term incubations. The common structural feature of compounds showing pro-oxidative effects in the short-term incubation was the presence of a methoxy group at position 3, and hydroxyl at position 5, and no other substituents attached to this ring, as seen for pinostilbene (**2**). Pinostilbene (**2**) was also the most pro-oxidative substance, increased the level of ROS by 60.4% in comparison to the control in the long-term incubation. Resveratrol (**1**) with a 36.9% increase of ROS was next, and pinostilbenoside (**7**), thunalbene (**3**) and batatasin III (**6**) with 3,5-dihydroxy, 2,4-dihydroxy, or 3,5-methoxy arrangements also increased the levels of ROS. The pro-oxidant effect shown by resveratrol (**1**) after long-term incubation and possibly also by other stilbenoids can be ascribed to H_2_O_2_ and other ROS formed as they decompose in culture medium [16]. This has not been observed during short-term exposures. Contrary to our results, Chao et al. found resveratrol (**1**) and pinostilbene (**2**) protecting neuron SH-SY5Y cells against oxidative stress caused by 6-hydroxydopamine [25].

Enzymatic systems that eliminate excessive ROS, such as CAT, GPx, and SOD, and enzymes that regenerate and produce endogenous antioxidants, such as glutathione reductase, thioredoxin reductase, or glutathione synthetase, make the most important contribution to the antioxidant defense of cells [26]. By increasing the expression of the aforementioned enzymes, natural phenolics exert an indirect antioxidant effect. It is possible that the increased expression of antioxidant enzymes is mediated by activation of the Keap1-Nrf2-ARE (Kelch-like ECH-associated protein 1-nuclear factor erythroid 2–related factor 2 (Nrf2)-antioxidant responsible elements) signalization pathway. Some phenolics are able, via Nrf2, to induce certain enzymes of phase 1 and phase 2 (e.g., glutathione-S-transferase, UDP-glucuronyltransferase) of metabolic elimination to detoxify pro-oxidant xenobiotics. [26,27]. Nrf2 is blocked in the cytoplasm by its inhibitor Keap1. Nrf2, activated by electrophilic stimuli, such as ROS, reactive nitrogen species (RNS), heavy metal ions, or some diseases) is released from the cytoplasmic protein Keap1 and translocated into nucleus, where it binds to the ARE area that regulates the expression of the target genes, for example, of HO-1, NAD(P)H:quinone oxidoreductase (NQO-1), or γ-glutamylcysteinsynthetase (γGCS), which increases the levels of glutathione [28,29,30].

Enzymes that cause antioxidant effects are activated by an increase in their responsible coding genes mediated by Nrf2. Most compounds that affect Nrf2 is thought to be pro-oxidants acting on Keap1, probably by reacting with sulfhydryl groups of cysteine. Nrf2 is then released [26]. Compared to the direct antioxidant effect of scavenging ROS (commonly observed using concentrations of 10–100 µM), the activity involving Keap1/Nrf2/ARE is more effective, requiring only 0.5–5 µM concentrations. It is independent of the stoichiometry and can amplify the initial direct effect of phenolics beyond the timeframe of direct activity, making them effective even after they are no longer present in situ [26].

Armed with this knowledge, we analyzed the effects of compounds **2**, **4**, and **18** on the expression of enzymes associated with oxidative stress. The effects of compounds **2**, **4**, and **18** on the levels of CAT, GPx, HO-1, and SOD-1 and -2 were evaluated after 6 h of incubation. The compound **2** decreased expression of CAT enzyme and basically unaffected expression of other proteins. Compound **18** elevated expression of Nrf2 significantly and CAT non-significantly. Standard quercetin significantly increased the expression of CAT and positive control pyocyanin upregulated HO-1. Greater concentrations of test compounds, or a different period of incubation, or both together might increase the effect. The effect of compound **18** on the expression of several enzymes associated with antioxidant defense was clearly visible, and we, therefore, analyzed effects of the test compounds **2**, **4** and **18** on the upstream regulation caused by Nrf2. As seen in Figure 7, compound **18** increased the activation of Nrf2-ARE system in a significant manner. This result corresponds to the elevated expression of Nrf2 and CAT.

Our results confirm previous studies showing that the stilbenoids, such as resveratrol (**1**) significantly affect the activity of Nrf2 and the levels of enzymes under its control. Resveratrol (**1**) enhances autophagy by increasing the activity of Nrf2. This activity is mediated by the binding of the adaptor protein p62, phosphorylated by kinases in the cytoplasm, to Keap1 in competition with Nrf2. The phosphorylated p62 binds to Keap1, releasing Nrf2, which translocates to the nucleus. [28,30]. Similar effects have been observed for oxyresveratrol [31]. Another stilbenoid able to increase the activity of Nrf2 is pterostilbene (**15**). A study by Saw et al. has shown, that pterostilbene (**15**) (and quercetin and kaempferol, two other phenols found in berries) directly scavenged ROS, activated the Nrf2-ARE signal pathway and acted synergistically when used together at some concentrations [19]. The incubation of cells with piceatannol (**4**) (10–20 μM) also increased the expression of HO-1. It is expected, that electrophilic quinone formed by the oxidation of piceatannol (**4**) binds directly to Keap1 and initiates this process [32]. The activity of cajanin stilbene acid may also be due to the effect on Nrf2 [33]. Similar effects were also seen in tests of extracts rich in stilbenoids, such as *Vitis vinifera* root extract which contains resveratrol (**1**) [29].

## 5. Conclusions

A group of several stilbenoids was investigated in vitro, using a cellular model of THP-1 macrophage-like cells to evaluate their antioxidant or pro-oxidant properties, to determine any effects that might possibly have on the expression of the enzymes catalase, glutathione peroxidase, and heme oxygenase-1, and their effects on the activation of Nrf2. The experimental results showed that these stilbenoids could affect the formation of reactive oxygen species in a cellular model, producing either an antioxidative or pro-oxidative effect, depending on the structure. Selected stilbenoids also affected the expression of antioxidant enzymes and showed some effects on the activation of Nrf2. Depending upon their structure, stilbenoids can possess either antioxidative or pro-oxidative effects, and they can also affect enzymatic antioxidant defenses. Pinostilbene (**2**) showed rather pro-oxidant effects and also decreased expression of CAT in cell culture. Piceatannol (**4**) had antioxidant effect as it directly scavenged ROS and isorhapontigenin (**18**) had a mild direct antioxidant effect, but on the other hand, activated Nrf2-ARE system and elevated expression of Nrf2 and CAT.

## Figures and Tables

**Table:** 

	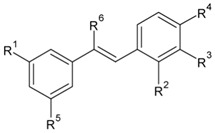						
		R^1^	R^2^	R^3^	R^4^	R^5^	R^6^
**1**	*Trans*-resveratrol	OH	H	H	OH	OH	H
**2**	Pinostilbene	OCH_3_	H	H	OH	OH	H
**3**	Thunalbene	OCH_3_	H	OH	H	OH	H
**4**	Piceatannol	OH	H	OH	OH	OH	H
**5**	Piceatannol-3´-*O*-β-glucopyranoside	OH	H	*O*-Glc	OH	OH	H
**7**	Pinostilbenoside	OCH_3_	H	H	*O*-Glc	OH	H
**11**	3,5-dimethoxystilbene	OCH_3_	H	H	H	OCH_3_	H
**12**	*Trans*-stilbene	H	H	H	H	H	H
**13**	*Cis*-stilbene	H	H	H	H	H	H
**14**	4-Stilbenecarboxylic acid	H	H	H	COOH	H	H
**15**	Pterostilbene	OCH_3_	H	H	OH	OCH_3_	H
**16**	*Trans*-α-methylstilbene	H	H	H	H	H	CH_3_
**17**	Pinosylvin monomethyl ether	OCH_3_	H	H	H	OH	H
**18**	Isorhapontigenin	OH	H	OCH_3_	OH	OH	H
**19**	2,4,3´,5´-tetramethoxystilbene	OCH_3_	OCH_3_	H	OCH_3_	OCH_3_	H

**Table:**
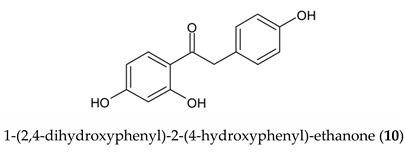


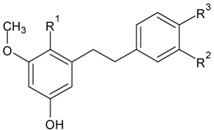				
		R^1^	R^2^	R^3^
**6**	Batatasin III	H	OH	H
**8**	2-carboxyl-3-*O*-methyl-4´-*β*-D-glucopyranosyl-dihydroresveratrol	COOH	H	*O*-Glc
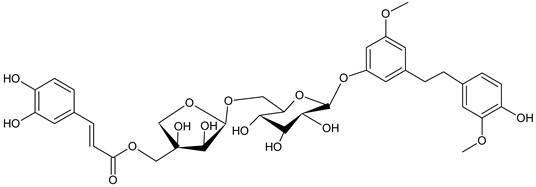 3-*O*-caffeoyl-(9→5)-β-apiosyl-(1→6)-β-glucopyranosyl-5,3´-*O*-dimethyldihydropiceatannol (**9**)				
